# Clinimetric properties of a smartphone application to measure the craniovertebral angle in different age groups and positions

**DOI:** 10.1016/j.heliyon.2023.e19336

**Published:** 2023-09-01

**Authors:** Aafreen, Ashfaque Khan, Ausaf Ahmad, Abdur Raheem Khan, Neeraj Maurya, Mohammed M. Alameer, Ahmad H. Hakamy, Mohammed A. Hakami, Yousef M. Alshehre, Mohammed Aljahni, Karthick Balasubramanian, Mohammed M. Alshehri, Mohammad Abu Shaphe

**Affiliations:** a Department of Physiotherapy, Integral University, Lucknow, India; b Department of Community Medicine, Integral University, Lucknow, India; c Physical Therapy Department King Fahad Central Hospital, Jazan, Saudi Arabia; d Medical Rehabilitation Center of King Fahad Central Hospital of Jazan, Saudi Arabia; e Physical Therapy Department, College of Applied Medical Sciences, Advance Rehabilitation Clinic, Jazan University, Jazan, Saudi Arabia; f Department of Physical Therapy, Faculty of Applied Medical Sciences, University of Tabuk, Saudi Arabia; g Physical Education Department, Jazan University, Jazan, Saudi Arabia

**Keywords:** surgimap system software, Surgimap smartphone application, Craniovertebral angle, Validity, Reliability

## Abstract

**Background:**

Craniovertebral angle (CVA) alteration is a causative factor for the neck, shoulder, and temporomandibular joints disorders. Therefore, as an outcome measure for therapeutic intervention, measuring the craniovertebral angle with the Surgimap smartphone app is a cost-effective, easily accessible, and reliable tool. This study's objective was to assess the clinimetric properties of the Surgimap smartphone application with Surgimap system software to measure the Craniovertebral Angle in different age groups and positions.

**Method:**

Ninety subjects with neck pain were randomly allocated to aged between 18 and 30 years (Group A; n = 45) and 45–60 years (Group B; n = 45). Using the Surgimap smartphone application and Surgimap system software, the craniovertebral angle was measured objectively in the sagittal plane. Intraclass correlation coefficients were used to determine validity and reliability. Receiver operating characteristic (ROC) curves and the area under the curves (AUC) were determined to distinguish participants with and without forward head posture.

**Result:**

The result of this study shows that Smartphone Surgimap Application and Surgimap System Software correlate 0.95 and have p-values of 0.01 for diverse positions and ages. CVA measurement in the sitting position was significantly lower than in the standing position, regardless of methodology or age. Both positions demonstrated high intra-rater reliability, as evidenced by Intraclass Correlation Coefficients (ICC) between 0.972 and 0.991. The minimum detectable change (MDC) values ranged from 1.3 to 1.733, indicating high measurement accuracy. The smartphone application demonstrated outstanding diagnostic sensitivity (100.00% for Group A standing) and specificity (93.55% for Group B standing).

**Conclusion:**

The Surgimap smartphone application is a reliable and accurate method for craniovertebral angle measurement and is useful for measuring outcomes. Also standing posture was found to be better than sitting posture while measuring the CVA.

## 1 Introduction

The optimal alignment of muscles and bones that minimizes the physical pressure and tension on the body is referred to as ideal posture. Among the most essential aspects of the musculoskeletal physical examination procedure is the postural assessment. Even in situations where proper posture is required, many people lack it [1]. The evaluation and adjustment of posture alignment have been correlated with enhanced health outcomes, energy efficiency, and mechanical benefit during a person's involvement in the practice. Health care professionals use several approaches to determine body orientation and posture imbalances [2]. These approaches vary from basic visual observation in clinical practice to more sophisticated laboratory-based motion analysis systems [3].

Postural angles are factors that could be calibrated for measuring posture. These are distinct from linear measures that represent posture variations as distances between two bony landmarks [4]. It is well known that angular measurements between body parts can be used to evaluate posture quantitatively [5]. There is a shortage of appropriate clinical quantitative assessment instruments to track changes in posture. For the purpose of assessing the outcome of therapeutic approaches, a valid and reliable examination of forward head posture (FHP) is necessary. For the purpose of assessing the outcome of therapeutic approaches, a valid and reliable examination of forward head posture (FHP) is necessary. Yip et al. found that there is no specific clinical procedure for calculating the craniovertebral angle accurately in people with or without cervical pain [6]. The Craniovertebral angle (CVA) is frequently used in assessing Forward Head Posture (FHP). Prior research has demonstrated its reliability and validity as an indicator of FHP severity [7]. The CVA is determined by the intersection of a horizontal line passing through the spinous process of the seventh cervical vertebra and a line passing through the tragus of the ear. A smaller CVA implies a more pronounced FHP [8]. If an individual's CVA is less than 48°, they are classified as having FHP, whereas a CVA greater than 48° indicates a normal craniovertebral posture. It has been stated that this procedure is very reliable (ICC = 0.88) [9,10]. The Surgimap System Software supports healthcare professionals in viewing, storing, and measuring photographs as well as in the planning of orthopaedic surgery. All the functionality of a modern laptop are presently available on smartphones, including online browsing, Wi-Fi, and third-party apps, etc. The most famous smartphones that are emerging today are Android, IOS smartphone operating systems. The popularity of smartphones is growing, and this can be utilized for better things, especially in the field of health care. In the medical sector, it is normal practise to use resources for examination, assessment, diagnosis, and follow-up [11].

Surgimap is a free smartphone application used for displaying, storing, and transporting images, and is much easier to manage. Surgimap Smartphone technology was used for radiological purposes by orthopaedics but was not historically used as an assessment method for photographic analysis of postural angles. The Surgimap Smartphone application is easily available, requires minimal training to operate, less time-consuming and simple-to-use tool to instantly analyse images inside a smartphone application; no instrumentation setup is needed for posture evaluation. The Surgimap smartphone application is intended for recreational use and can calculate various angles; however, there are no records of its reliability and validity measurements to date. Thus, in the current study, we examine the clinimetric properties of the smartphone Surgimap application for calculating CVA in sitting and standing positions for different age groups.

## 2 Methodology

We conducted the study after obtaining ethical clearance from the Institutional Ethical Committee (IEC/IIMS&R/2022/70) of Integral University, Lucknow, India. The population included in this study was a convenient sample, recruited by purposive and snowball sampling at Physiotherapy OPD, Integral Hospital and Research Centre, Lucknow. The sample size was calculated using the G. Power 3.15 software developed by Franz F at Universität Kiel in Kiel, Germany. To ensure a minimum acceptable Intraclass Correlation Coefficient (ICC) value of 0.60 and a reliability of 0.75 (1-β = 0.80; α = 0.05) at a 95% confidence level, a minimum of 85 subjects were needed.

## 3 Participants

In this study, a total of 90 participants with neck pain were recruited and divided into two groups: Group A consisted of 45 participants aged between 18 and 30 years, while Group B consisted of 45 participants aged between 45 and 60 years. The participants were selected regardless of whether they had received professional treatment for their pain, but they were required to report a perceived neck pain intensity rating between 2-to-5 on a 0-to-10 visual analogue pain rating scale, and this rating could not increase by more than 2 during the data collection period. Both male and female participants were included, but those with a history of previous surgery of the cervical spine, neurological symptoms, severe spinal pathology, fracture, or disc herniation, psychiatric condition (such as dementia, amnesia, or delirium) or neurological disease (such as Multiple sclerosis or Lou Gehrig's Disease) were excluded. The study was conducted in accordance with the 1964 Helsinki Declaration, and all participants provided written consent before taking part in the study.

### 3.1 Instrumentation

Surgimap is digitising software developed for medical professionals. The programme is free and is used to calibrate and save the images. Surgimap is a free mobile application downloaded from the smartphone application store (Nemaris Inc., New York, USA). The digital still camera used was a Sony DSC-H20 (Cyber Shot; 10.1 megapixels, 10′ optical zoom; Sony Corp., Japan). Surgimap, a free computer digitising software downloaded from http://www.Surgimap.com (Nemaris Inc., New York, USA), was used to measure Cranio Vertebral Angle in the male subjects.

### 3.2 Procedure

Craniovertebral Angle (CVA) was measured for each subject of Group A and Group B in sitting as well as standing position. Prior to measurement, the subject removed eyeglasses, hats, and lifted and fastened any hair that were covering the ears, neck, or eyes. The subjects in both the groups chose the first position for measurement (either sitting or standing) by lottery methods [12]. Ninety folded sheets of the same size and form were kept in a box with 45 identified as belonging to sitting position and 45 as standing position. Paper drawn by the patient allocated the position for measurement to be chosen first.

### 3.3 CVA measurement of intrarater reliability

**Fig. 1:**
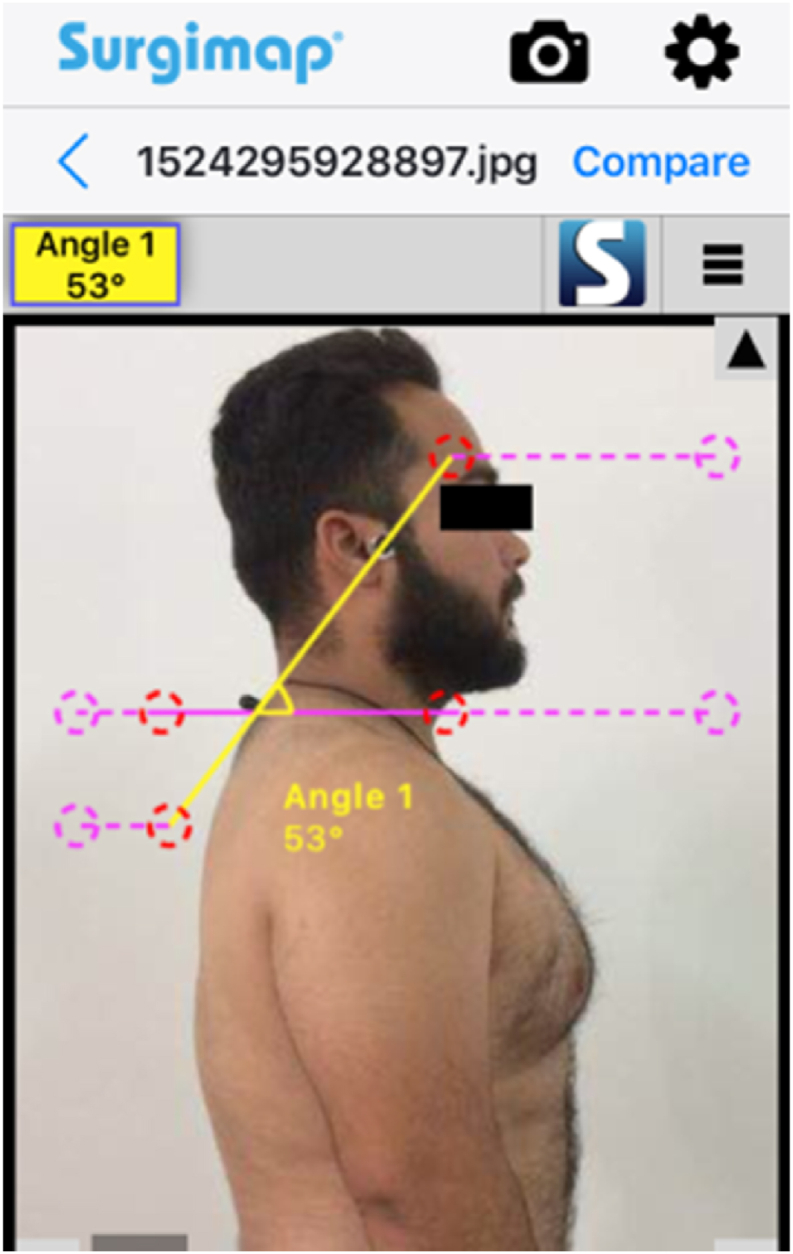
Measurement of CVA with Surgimap smartphone application in standing.

An image from the sagittal plane of each subject was taken at each session to provide objective access to CVA via a smartphone camera with a Surgimap application used for the analysis of images. Three measurements for intrarater reliability were taken on same days with 5 min rest period for each subject. The subject was positioned 1.5 m away from the smartphone camera. To standardise the subjects' head and cervical posture, a self-balancing pose was selected, and the height of the smartphone was set to the level of the participant's shoulder [13]. To achieve this position, the subject moves his cervical spine into full flexion and extension range and gradually reduces its range until the subject stops moving and holds his head and cervical spine in a neutral position. Subjects were instructed to maintain a neutral position until the photograph was taken [14]. For readings to be recorded in standing position, participants in both the groups were ordered to stand comfortably with their weight evenly distributed on both feet close to the plumb line suspended from the ceiling, the plumb line represents the true vertical line (Fig. 1), whereas for readings to be recorded in sitting position the subjects were instructed to sit on the chair with the back supported and the arm supported on the armrest of the chair, the coxo femoral and knee joints should be 90° flexed with the foot completely supported (Fig. 3). Participants were also instructed to keep natural head posture and focus eye directly on a focal point ahead on the wall in both positions to avoid difference in head position. Clothing was rearranged so that neck (C7 spine) and shoulders are exposed. The anatomical reflective adhesive marker was positioned over the tragus of ear and the spinous process of vertebrae prominence [15,16]. The examiner verified that the ears were visible during the photography. Once the image has been obtained, the picture was analysed to measure CVA using the Surgimap mobile application. In order to quantify CVA, the angle formed by the horizontal line through the spinous process of vertebrae prominence and the line connecting the ear tragus to the spinous process of vertebrae prominence was calculated. The CVA was determined with the Mobile Surgimap application for both groups A and B.

### 3.4 CVA validity measurement

**Fig. 2:**
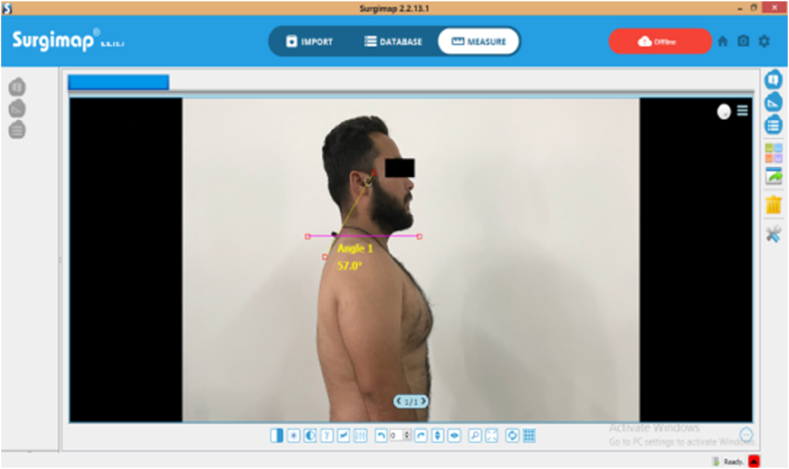
Measurement of CVA with Surgimap software in standing.

An image of the sagittal plane for each subject was captured with objective access to the CVA, the digital camera was positioned at 1.5 m and fixed with a camera stand without any rotation or tilt. In order to standardise the subject's head and neck position, the level of the camera was set to the height of the subject's shoulder and the image obtained was analysed using the Surgimap system software (Fig. 2, Fig. 4). The image taken by smartphone and camera at the same time and same position of the patient, first with smartphone then with the camera.

**Fig. 3:**
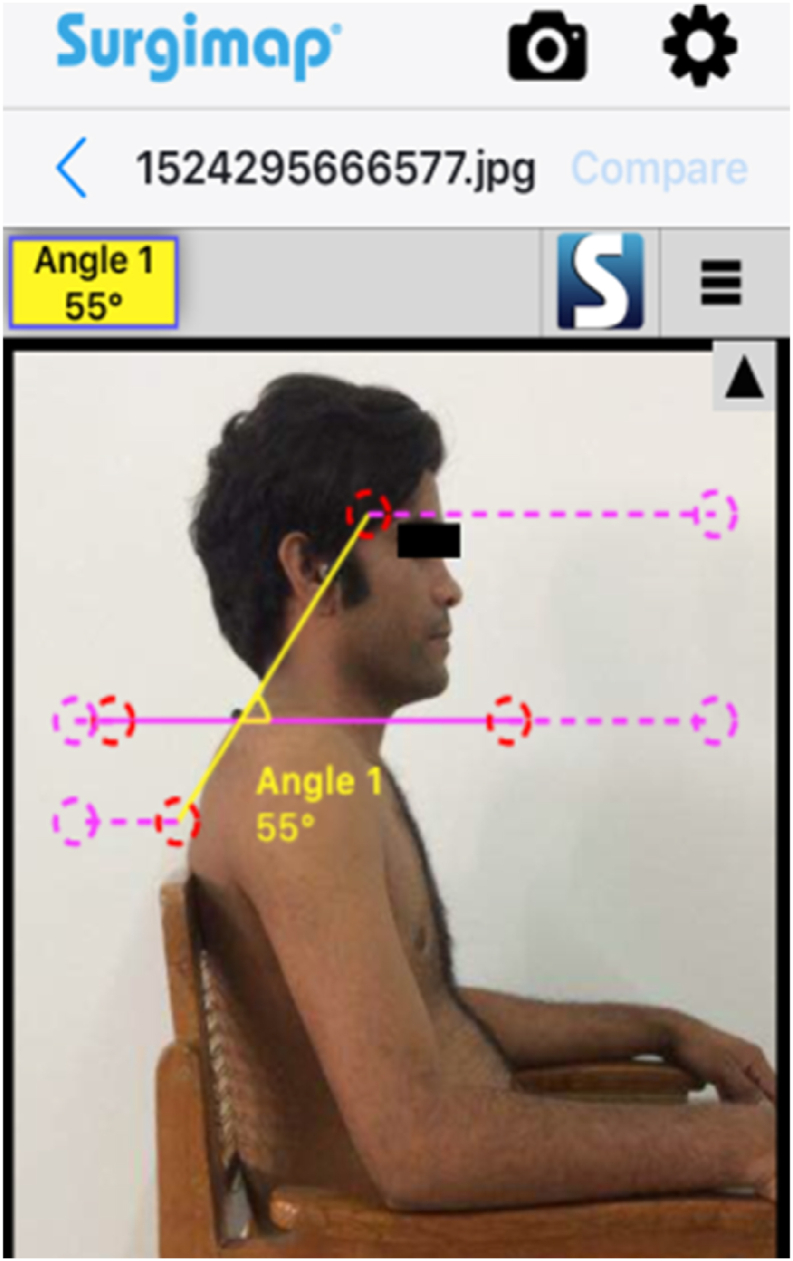
Measurement of CVA with Surgimap smartphone application in sitting.

**Fig. 4:**
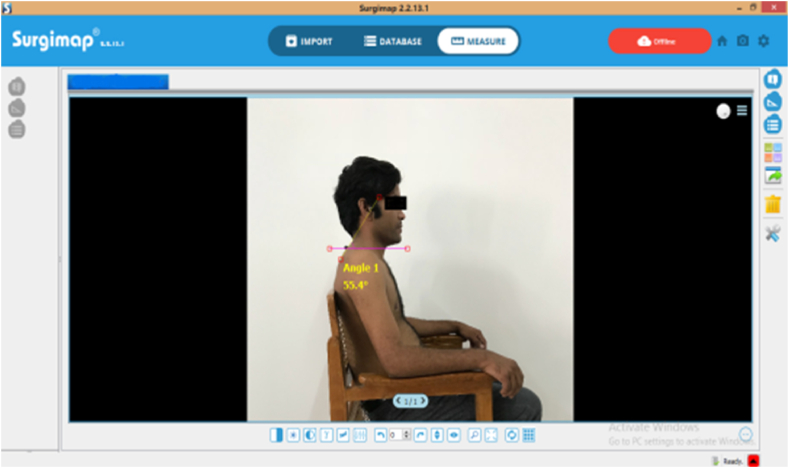
Measurement of CVA with Surgimap software in sitting.

### 3.5 Statistical analysis

A data statistical analysis was performed using SPSS 16.0 statistical software (SPSS, Chicago, IL, USA). For the general characteristics of the participants (i.e., age, gender, height, weight, and BMI), descriptive statistics were used. The mean and SD were calculated for continuous data. To establish the normal distribution of each variable, a Kolmograph-Smirnov (K–S) test was conducted. Subsequently, an independent *t*-test for continuous variable and chi square test were employed to compare the variables between the surgimap smartphone application and the surgimap system software across various positions. To evaluate the consistency of measurements, the interclass correlation coefficient (ICC) and its 95% confidence intervals (CIs) were used to determine the intrarater reliability. The Interclass Correlation Coefficient (ICC) calculation was used with a 95% CI accompanied by the standard error of the mean (SEM) SEM = SD √(1 – ICC)·ICC values above 0.75 indicated excellent reproducibility, while values ranging between 0.40 and 0.75 represented fair to good reliability, and values below 0.40 indicated poor reproducibility. To assess the measurement variability, the standard error of measurement (SEM) was computed using the mean square error term (WMS) from the analysis of variance. The SEM provides an absolute measure of reliability that represents the typical error associated with a measurement. Additionally, the minimal detectable change (MDC) equation was used to calculate the smallest amount of change that can be detected by a measure corresponding to clinically significant changes. These measurements were accompanied by the minimal detectable change (MDC) with the formula MDC = 1.96 √ 2 SEM. To see the correlation between readings from the Smartphone Surgimap Application (R1, R2, and R3) versus readings from the Surgimap System Software (R), Pearson correlation (r value) was used. An r = 0–0.2 indicated a very low correlation, probably meaning less; r = 0.2–0.4 indicated a low correlation; r = 0.4–0.6 indicated a reasonable correlation; r = 0.6–0.8 indicated a high correlation; r = 0.8–1.0 indicated a very high correlation. Bland-Altman plots, which include a 95% confidence interval, visualize the level of agreement between the Smartphone Surgimap Application and the Surgimap System Software in both Group A and Group B. Using this method, it plots the difference between each pair of measurements against their mean. The consistency between the Smartphone Surgimap Application and the Surgimap System Software in both Groups can be seen in this difference. A standard error of the mean is also shown to assess absolute reliability - how much-repeated measurements vary for an individual under the same circumstances.

The level of statistical significance was defined as a *p*-value less than 0.05. A ROC (receive operating characteristic curve) and a contingency table were used to figure out the sensitivity and specificity of the smartphone app. Accuracy was assessed using the area under curve (AUC). Values from 0.50 to 0.70 indicate low precision, 0.70 to 0.90 moderate precision, and higher than 0.90 high precision.

## 4 Results

The study recruited a total of 90 participants who were divided into two groups based on their age and neck pain. Group A comprised 45 participants aged between 18 and 30 years, consisting of 22 women and 23 men, and had a visual analogue pain scale rating of 4.13 ± 0.89. Group B consisted of 45 participants aged between 45 and 60 years, consisting of 23 women and 22 men, with a visual analogue pain scale rating of 3.62 ± 1.03. Table 1 shows the demographic data of the participants. The findings indicate a significant difference between the two groups concerning their BMI and VAS scale, whereas there was no significant difference in the gender distribution. .

**Table 1:** Distribution of Gender, BMI, and VAS scale of study subjects in both groups.

Characteristics		Group A	Group B	*p* value
BMI^#^		23.73 ± 0.98	24.32 ± 1.59	0.03*
VAS^#^		4.13 ± 0.89	3.62 ± 1.03	0.01*
**Sex**^**@**^	**Female**	22 (48.9%)	23 (51.1%)	0.83
	**Male**	23 (51.1%)	22 (48.9%)	

*P value < 0.05, considered as statistically significant; @: Chi square test; #: Independent test; VAS: Visual Analogue Scale; BMI: Body Mass Index.

The mean CVA values (± standard deviation) for each position and group are presented in Table 2. The results show that for both tools and both groups, the CVA values were significantly lower in the sitting position than in the standing position (*p* value = 0.00 for both).

**Table 2:** Comparison of CVA between different positions also in between groups.

Characteristics	Position	Group A	Group B	*p* value
Smartphone Surgimap	Standing	53.72 ± 2.11	51.58 ± 2.48	0.00*
	Sitting	56.35 ± 2.28	54.49 ± 2.40	0.00*
	***p* value**	0.00*	0.00*	
Surgimap System Software	Standing	53.76 ± 2.15	51.71 ± 2.53	0.00*
	Sitting	56.40 ± 2.30	54.62 ± 2.60	0.00*
	***p* value**	0.00*	0.00*	

*P value < 0.05, considered as statistically significant.

Table 3 presents the Pearson correlation coefficients and p-values between the readings from the Smartphone Surgimap Application (R1, R2, and R3) and the reading from the Surgimap System Software (R) for both standing and sitting positions. The table indicates a very strong positive correlation between the readings from the Smartphone Surgimap Application and the reading from the Surgimap System Software for both standing and sitting positions. All the correlation coefficients are above 0.95, indicating a very strong linear relationship between the two sets of measurements. Moreover, the p-values for all the correlations are 0.00, which indicates that the correlations are statistically significant, and the observed relationships are unlikely due to chance.

**Table 3:** Correlation between readings from Smartphone Surgimap Application (R1, R2, R3) versus reading from Surgimap System Software (R).

Readings from Smartphone Surgimap Application (R1, R2, R3) versus reading from Surgimap System Software (R)		Pearson Correlation (r value)	*p* value
Standing	R1 Vs R	0.971	0.00*
	R2 Vs R	0.980	0.00*
	R3 Vs R	0.962	0.00*
Sitting	R1 Vs R	0.979	0.00*
	R2 Vs R	0.970	0.00*
	R3 Vs R	0.955	0.00*

*P value < 0.05, considered as statistically significant.

**Fig. 5:**
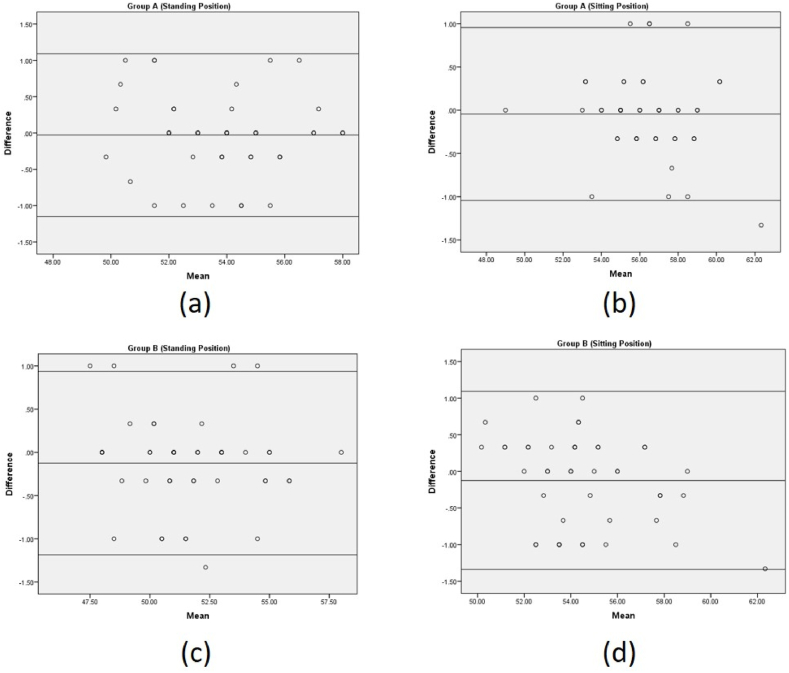
Bland and Altman chart for intra-rater reliability (a) Group A standing position (b) Group A sitting position (c) Group B standing position (d) Group B sitting position

The ICC values for all comparisons are very high, ranging from 0.972 to 0.990, indicating excellent agreement between the measurements obtained using the two systems. The SEM values for the comparisons range from 0.253 to 0.351, which indicates the amount of error in the measurement that can be attributed to random error. The minimum detectable change (MDC) reflects the smallest amount of change that can be detected with a particular measurement method. The MDC values for the comparisons range from 1.395 to 1.642, which is the smallest change that can be considered real and beyond the measurement error (presented in Table 4). Table 4 and Fig. 5 shows that there is very good intrarater reliability between the Smartphone Surgimap Application and the Surgimap System Software in both Group A and Group B in the standing position.

**Table 4:** The intrarater reliability of the standing position measurements obtained by the Smartphone Surgimap Application and Surgimap System Software.

Readings from Smartphone Surgimap Application (R1, R2, R3) versus reading from Surgimap System Software (R)		ICC	95% CI		SEM	MDC
			Lower	Upper		
Group A Standing	R1 Vs R	.978	.959	.988	0.320	1.569
	R2 Vs R	.985	.973	.992	0.261	1.417
	R3 Vs R	.972	.948	.984	0.351	1.642
Group B Standing	R1 Vs R	.986	.974	.992	0.297	1.510
	R2 Vs R	.990	.981	.994	0.253	1.395
	R3 Vs R	.980	.964	.989	0.348	1.636

ICC: intraclass correlation coefficient; CI: confidence interval; SEM: standard error of measurement; MDC: minimum detectable change.

Table 5 presents the intrarater reliability of the Smartphone Surgimap Application versus the Surgimap System Software in the sitting position for two groups, A and B. The intraclass correlation coefficient (ICC) is used as a measure of agreement between the two measurement methods. The ICC ranges from 0 to 1, with values closer to 1 indicating higher agreement. The ICC values are high and range from 0.972 to 0.991, indicating excellent agreement between the readings from the Smartphone Surgimap Application and Surgimap System Software. The 95% confidence intervals (CI) for the ICC values are relatively narrow, ranging from 0.949 to 0.995, which suggests that the estimates are relatively precise. The SEM values range from 0.220 to 0.391 for sitting position, indicating that the measurements are relatively precise. The MDC values in sitting position range from 1.3 to 1.733, which suggests that changes greater than this amount can be detected with confidence.

**Table 5:** The intrarater reliability of the sitting position measurements obtained by the Smartphone Surgimap Application and Surgimap System Software.

Readings from Smartphone Surgimap Application (R1, R2, R3) versus reading from Surgimap System Software (R)		ICC	95% CI		SEM	MDC
			Lower	Upper		
Group A Sitting	R1 Vs R	.991	.983	.995	0.220	1.300
	R2 Vs R	.987	.977	.993	0.259	1.410
	R3 Vs R	.972	.949	.985	0.391	1.733
Group B Sitting	R1 Vs R	.985	.973	.992	0.303	1.525
	R2 Vs R	.977	.959	.988	0.365	1.675
	R3 Vs R	.974	.953	.986	0.388	1.727

ICC: intraclass correlation coefficient; CI: confidence interval; SEM: standard error of measurement; MDC: minimum detectable change.

Table 6 represents that the smartphone application exhibited the higher accuracy in terms of sensitivity and specificity for both the groups in standing position.

**Table 6:** Sensitivity, specificity, PPV, NPV and Accuracy of reading in different position for both groups.

	Group	Sensitivity	Specificity	PPV	NPV	Cut-off	Accuracy
Standing	A	100.00%	93.18%	25.00%	100.00%	66.67	93.33%
	B	85.71%	93.55%	85.71%	93.55%	61.55	91.11%
Sitting	A	83.33%	89.74%	55.56%	97.22%	59.62	88.89%
	B	80.00%	85.00%	40.00%	97.14%	62.54	84.44%

PPV: Positive Predictive Value; NPV: Negative Predictive Value.

In the standing position, Group A demonstrated a sensitivity of 100.00%, indicating that it correctly identified all positive cases. The overall accuracy of reading in the standing position for Group A was 93.33%. In the standing position for Group B, the sensitivity was 85.71%, suggesting a high proportion of correctly identified positive cases. The accuracy of reading in the standing position for Group B was 91.11%. In the sitting position, Group A demonstrated a sensitivity of 83.33%, indicating a relatively high proportion of correctly identified positive cases. The accuracy of reading in the sitting position for Group A was 88.89%. In the sitting position for Group B, the sensitivity was 80.00%, suggesting a relatively high proportion of correctly identified positive cases. The accuracy of reading in the sitting position for Group B was 84.44%.

**Fig. 6:**
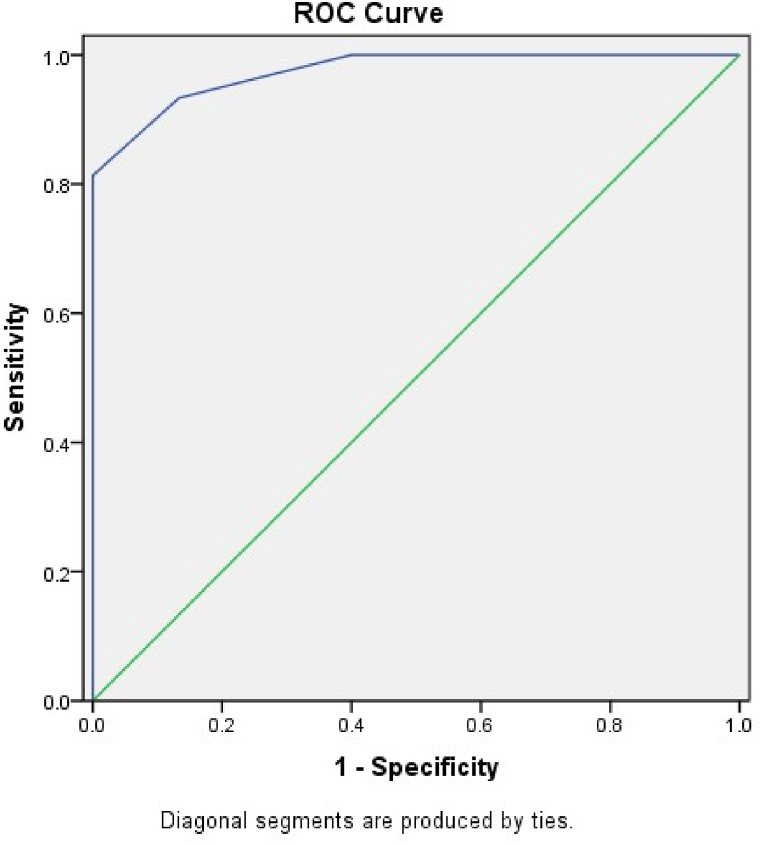
ROC (Receiver Operating Characteristic) curve at standing position.

**Fig. 7:**
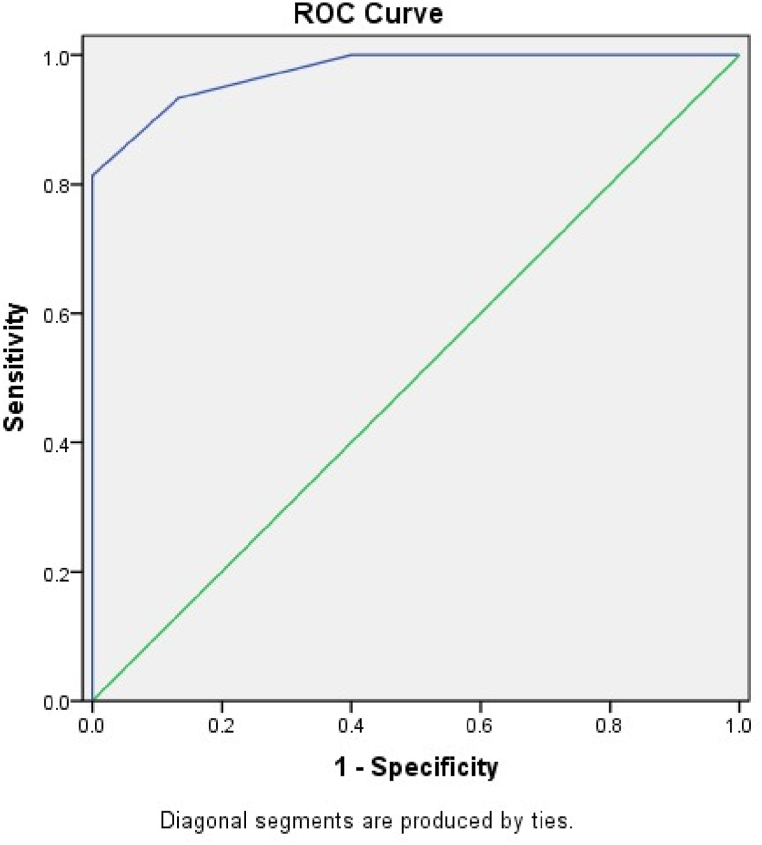
ROC (Receiver Operating Characteristic) curve at sitting position.

The ROC curve in Fig. 6 shows the performance of a predictive model in distinguishing between two classes (possibly healthy and diseased) at the standing position. The AUC (Area under the Curve) is a summary statistic that measures the overall performance of the model. The AUC value of 0.974 and 0.963 indicates that the model has high discriminative ability and can effectively distinguish between the two classes in both postures respectively. The standard error of 0.015 and 0.013 and p-value of 0.000 and 0.000 suggest that the observed AUC is statistically significant and unlikely to be due to chance. The 95% confidence interval of 0.945–1.000 indicates and 0.934 to 0.0989 that the true AUC lies within this range with 95% confidence. Overall, the high AUC value, significant p-value, and narrow confidence interval suggest that the predictive model performs well at distinguishing between the two classes at the standing position as well as in sitting position (Fig. 7).

## 5 Discussion

When assessing the physical state and function of the cervical spine, reliable and valid instruments that provide accurate and consistent measurements of the craniovertebral angle are crucial. The current study revealed that the intra-class reliability of the smartphone Surgimap application showed excellent results, with an ICC value above the 0.9 and the Surgimap mobile application is proven to be a reliable measurement tool for craniovertebral angle in participants with neck pain in different age groups and positions. Digital photography has become increasingly common, but it is not yet a universal standard of care. When measuring photographic measurements, consideration should also be given to the impact of using instruments such as the Smartphone Surgimap application. Similar findings were found by Niekerk et al. when the images were utilized to determine the location of the head, shoulder, and chest [17]. Also, Iunes have observed similar findings when measuring global posture in standing [18]. One of the previous studies examined the significance of digitization methods and the application of reflective markers for posture evaluation and found ICCs ranging from inappropriate to appropriate for posture parameters [3]. The study also showed construct validity when digitising photos using a mobile posture application for both segment translations and angulations, as well as good to excellent intra- and inter-rater reliability. In comparison to goniometric or inclinometer readings, it was observed that the mobile app offered more posture-related variables, were more user-friendly and cost-effective [19].

Both a smartphone application and software measure the angle in the same way, however the smartphone is portable, whereas the programme requires either a computer setup or a laptop. In the present research, a smartphone application called Surgimap was formulated, which can be used as a gold standard CVA measurement tool. The introduction of smartphones has made a variety of clinical measuring applications accessible to most clinicians. Most smartphones contain built-in sensors such as accelerometers, magnetometers, and gyroscopes that enable them to find the joint position and measure joint ROM [20]. Moreover, mobile applications have emerged as a potential substitute for photogrammetry devices since they streamline image acquisition and data analysis while also lowering expenses [21].

Smartphone-based Surgimap application measurement techniques may be used to open a photographed or scanned image that forms a digitized image. It changes the contrast and brightness of the image to allow a better understanding of important anatomical parameters that are not typically measurable in the conventional photographic process. The measurement technique of the Surgimap smartphone application has some benefits, such as, a fast comparison of images collected at various intervals of the patient, and affordable storage. In the current study, there was no statistically significant difference in intrarater reliability between the Surgimap smartphone application and the Surgimap system software for CVA measurement. Previously, Surgimap programme is a reliable tool for calculating adolescent vertebral postural angles from different viewpoints in standing posture from digital images [22]. Another study concluded that the Surgimap programme calculation is an equal measuring method to the conventional coronal cobb angle manual, but is beneficial for spinopelvic measurements in T2-T5, pelvic incidence, sacral slope, pelvic tilt, lumbar lordosis [23]. Research concluded that to examine head and cervical posture, the photogrammetric approach exhibited great inter and intra rater reliability and also in order to differentiate between females with moderate-severe and non-FHP, the craniovertebral angle approach is more accurate when compared to head position angle and head tilt angle [24].

The study showed that the Surgimap smartphone application is significantly reliable and valid for the calculation of the craniovertebral angle and can be used as a convenient method for physical therapists for a posture assessment. We assessed the reliability and validity of the Surgimap smartphone application for the calculation of the craniovertebral angle. It also determined that there is a significant difference between smartphone readings and system software reading for craniovertebral angle measurement in the two different positions, i.e., standing and sitting. It states that standing posture is more significant than sitting posture while measuring craniovertebral angle. Fernandez-de-Las-Penas et al. found no significant alterations in the measurements of forward head posture (FHP) during relaxed sitting and relaxed standing positions, as per the standardized protocol. However, this outcome might be attributed to the lack of usage of a plumb line to accurately determine the true horizontal line across C7 [13].

The results showed that CVA values were significantly lower in the sitting position than in the standing position for both groups, indicating that sitting for prolonged periods can lead to greater forward head posture and increased cervical spine flexion, contributing to neck pain and discomfort. Although CVA values were consistently higher in Group A than in Group B for both positions and tools, the difference was not statistically significant. The lack of significant differences in CVA values between the two age groups is consistent with previous studies that found no significant differences in CVA values between different age groups [25,26].

It has been observed that different sitting and standing postures have an impact on dorso-lumbar spinal posture, head, cervical posture, and motor function [27]. The subjects were instructed to lean on a backrest while sitting comfortably, which could result in a posterior pelvic tilt and a flat back posture. Contrarily, when a thoracic support is used, dorsal spine and trunk are pushed forward, causing the spine to slant toward the dorsal spine kyphosis. In this case, the head might be tilted forward and down. But because eyes naturally look at eye level, the head has to be raised from there, which causes FHP [28]. When the head is held upright and above the feet, the lower limbs and all of the axial skeleton's segments function like links in a connected chain. Movement in one chain link induces at least one other chain link to move [29]. The above-mentioned changes could be the reason for more accurate assessment of CVA in standing position.

The study's strength lies in its use of two different tools to measure CVA values, which increases the study's reliability and validity. The study's limitations include firstly its small sample size and the absence of a control group. Second, we have not checked the interrater reliability of the smartphone Surgimap application. Furthermore, the study did not examine the effect of interventions or treatments on CVA values, which limits the study's generalizability to clinical practice.

## 6 Conclusion

It is clear from the analysis of the current study that there is a strong correlation of craniovertebral angle measured by smartphone Surgimap application and Surgimap system software which supports that smartphone Surgimap application can be recommended and is a reliable and valid tool for clinical evaluation and examination of craniovertebral angle. Also, this study provides valuable information on the relationship between sitting and standing positions and CVA values in participants with neck pain in different age groups using two different measurement tools. The study highlights the importance of avoiding prolonged sitting to prevent forward head posture and increased cervical spine flexion, which can contribute to neck pain and discomfort. With increasing demand being placed on evidence-based practice, a valid and reliable outcome measuring tool should contribute to better clinical service and evaluation. Clinicians always try to correct their patients' forward head postures by various treatment approaches. It is essential to create a method that can measure patients' posture objectively in order to evaluate the efficacy of various approaches. A practical, user-friendly, reliable, and objectively measuring devices should be used. The surgimap smartphone app fulfils the aforementioned function because it is a low-cost, portable, and practical tool that enables clinicians to gather a reliable, precise, and objective reading. The instrument provides clinicians with additional useful and reliable information to monitor patients’ condition and progression. Future research should investigate the effect of interventions or treatments on CVA values in participants with neck pain.

## Author contribution statement

1 - Conceived and designed the experiments;

2 - Performed the experiments;

3 - Analysed and interpreted the data;

4 - Contributed reagents, materials, analysis tools or data;

5 - Wrote the paper.

## Funding statement

The authors extend their appreciation to the Deputyship Ministry of Education in Saudi Arabia for funding this research work ISP22-7.

## Data availability statement

Data will be made available on request.

## Declaration of competing interest

The authors declare that they have no known competing financial interests or personal relationships that could have appeared to influence the work reported in this paper.
